# The profile of inflammatory extracellular vesicles in intracerebral hemorrhage patients

**DOI:** 10.3389/fstro.2022.988081

**Published:** 2022-09-20

**Authors:** Harshal Sawant, Trevor Bihl, Doan Nguyen, Ifeanyi Iwuchukwu, Ji Bihl

**Affiliations:** ^1^Department of Biomedical Sciences, Marshall University, Huntington, WV, United States; ^2^Department of Pharmacology and Toxicology, Wright State University, Dayton, OH, United States; ^3^Institute for Translational Research, Ochsner Medical Center, New Orleans, LA, United States; ^4^Department of Neurology, Ochsner Medical Center, New Orleans, LA, United States

**Keywords:** intracerebral hemorrhage, extracellular vesicles, large extracellular vesicles, small extracellular vesicles, microgila, neutrophil, macrophage

## Abstract

**Background:**

Intracerebral hemorrhage (ICH) is one of the leading life-threatening types of strokes with high mortality. A prominent feature of ICH is neuroinflammation involving leukocytes, such as neutrophils and macrophages. Large extracellular vesicles (lEV) and small extracellular vesicles (sEV) released from various cells are used as biomarkers for different diseases. Here, we aimed to determine the concentration/population of lEV and sEV from different leukocytes in ICH patients and analyze the correlation of these lEV/sEV with clinical parameters.

**Methods:**

lEV and sEV were isolated from the plasma of ICH patients (n = 39) by using the serial centrifuge methods. Nanoparticle tracking analysis (NTA, NS300) was used to determine the type and concentration of different leukocytes-released lEV/sEV. Specific antibodies, CD66b, P2RY12, and CD80 were used for different leukocyte types.

**Results:**

A predictive relationship between both hospital length of stay (*R*^2^ = 0.83) and Intensive care units (ICU) length of stay (*R*^2^ = 0.88) was found with lEV and sEV and patient data [including low-density lipoprotein (LDL), ICH volume, etc.]. Further predictive multiple linear regression relationship was seen between lEV and sEV concentrations and MRSV3 (Modified Rankin Scale at 90 days) (*R*^2^ = 0.46) and MRSV5 (modified Rankin Scale at 180 days) (*R*^2^ = 0.51). Additionally, a slight, but statistically significant (*p* = 0.0151), multiple linear regression relationship was seen between lEV and sEV concentrations and ICU length of stay (*R*^2^ = 0.26).

**Conclusion:**

This study found predictive relationships between patient outcomes and lEV and sEV. When combined with generally collected patient data (LDL, etc.), measurements of lEV and sEV are strongly predictive of overall patient outcome. Further, larger studies should investigate these effects.

## Introduction

Intracerebral hemorrhage (ICH) is a type of stroke characterized by spontaneous and non-traumatic bleeding in the brain. ICH constitutes 15–20% of strokes but is particularly catastrophic with a mortality rate of up to 50% (Xue and Yong, 2020). When ICH occurs, blood collects in the brain and causes brain damage, neuronal death, and varying degrees of functional impairments, associated with neuroinflammation. ICH is associated with hypertension and may also be caused by coagulopathy, cerebral amyloid angiopathy, brain tumors, vascular anomalies, brain trauma, or premature birth. Despite the clinical advances of surgical procedures that are minimally invasive to remove the clot (Gross et al., 2019), the prognosis of ICH is poor because of the combination of primary and secondary injury. Primary injury includes mechanical disruption from extravagated blood and hematoma expansion. Secondary injury soon develops and is driven by excitotoxicity, edema and oxidative stress (Wan et al., 2019). Crucially, a cascade of inflammatory processes occurs around the hematoma (Xue and Yong, 2020).

A prominent feature of ICH is neuroinflammation, particularly the excessive representation of pro-inflammatory central nervous system (CNS) intrinsic microglia and monocyte-derived macrophages that infiltrate from the circulation (Lan et al., 2017; Bai et al., 2020). Immediately following the primary injury with a pronounced death of neurons, there is the release of damage-associated molecular patterns from degenerating neurons and the neuropil. Together with a chemokine gradient, the elevated vascular adhesive proteins trap leucocytes, particularly neutrophils that then transmigrate into the CNS parenchyma within the early minutes to hours of injury; lymphocytes enter hours to days later. Monocytes in the circulation are recruited in the early hours of ICH, and they mature into macrophages in the CNS parenchyma (Lan et al., 2017; Shao et al., 2019; Shi et al., 2019; Bai et al., 2020).

Microglia constitute 5–10% of the total cellular population within the normal brain; they act as the first and main form of active immune defense intrinsic to the CNS (Poon et al., 2017). In response to pathology signals, they can change morphologically and functionally, similar to macrophages. The pro-inflammatory macrophages (M1) produce injury-enhancing factors, including inflammatory cytokines, matrix metalloproteinases and reactive oxygen species. Conversely, the regulatory macrophages (M2) exhibit potential reparative and anti-inflammatory roles. Both macrophages contribute to pro-inflammatory and homeostatic mechanisms within the brain through the secretion of cytokines and other signaling molecules (Mishra and Yong, 2016; Bai et al., 2020).

Cells release two types of extracellular vesicles (EVs): large extracellular vesicles (lEV, 120–1000 nm) and small extracellular vesicles (sEV, 30–120 nm) (Bister et al., 2020). EVs can be secreted by almost all cells, which have been recognized as a novel platform for intercellular communication in the CNS (Gassama and Favereaux, 2021; Jin Q. et al., 2021). They are also considered an important player in the tissue “microenvironment” (Losurdo and Grilli, 2020; Busatto et al., 2021) and inter-organ communications (Venkat et al., 2018; Yerrapragada and Bihl, 2021). EVs are capable of transferring proteins, lipids and nucleic acids between cells in the brain (e.g., neurons and microglia, astrocytes and neurons), contributing to CNS development and maintenance of homeostasis. Evidence shows that EVs originating from CNS cells act as suppressors or promoters in the initiation and progression of neurological disorders (Jin Q. et al., 2021). Moreover, these EVs have been shown to transfer molecules associated with diseases through the blood-brain barrier (BBB) and thus, can be detected in blood (Chen et al., 2017; Venkat et al., 2018). This unique feature enables EVs to act as potential diagnostic biomarkers for neurological disorders. In addition, other and our previous research showed that sEV (e.g., exosomes) from stem cells provide therapeutic effects on ischemic stroke (Xin et al., 2017; Chen and Chopp, 2018; Wang et al., 2020). We also have discovered that circulating endothelial lEV/sEV could be the biomarker for ischemic stroke patients (Wang et al., 2016a). However, there is no research found to identify the cell origin or the population of different leukocytes of lEV/sEV that is involved in the neuroinflammation and define them as biomarkers of brain damage in ICH.

The present study aimed to determine the level and phenotype of circulating lEV/sEV of ICH patients and to identify the correlation of circulating lEV/sEV level with the outcome of ICH. Specifically, the levels of CD66b+ lEV/sEV, P2RY12+ lEV/sEV, and CD80+ lEV/sEV in the circulation were measured. Moreover, the correlation of the concentration of lEV/sEV with the outcomes and severity of ICH was analyzed.

## Methods

### Patient information

A total of 39 patients (23–79 years) with ICH were included in this study. 22 of them were males, 17 were females and a total of 15 were 58 years old. Patients were recruited from the Neurology Department of Ochsner Medical Center (OMC), New Orleans, LA. The protocol was approved by the Marshall University and Ochsner Medical Center's institutional review board (IRB, #2015.137.A). Each patient signed an informed consent after a thorough explanation. Briefly, eligible patients are approached within 48 h from symptom onset of ICH confirmed on neuroimaging (computed tomography brain scan) at OMC. Dr. Ifeanyi Iwuchukwu at OMC discusses participation and obtains informed consent. Inclusion and exclusion criteria are described in Table 1. Specifically, enrollment and sample collection occur once informed consent is obtained.

**Table 1:** Inclusion and exclusion criteria.

**Inclusion**	**Exclusion**
Age ≥18 years	Malignancy, autoimmune disease, trauma, vascular malformation
CT or MRI diagnosis of acute ICH	Recent ischemic or hemorrhagic stroke within 3 months
Onset or last known normal <48 h	Pregnancy
	Sepsis on admission or recent infection within 3 months
	History of pancreatitis, cholecystitis, or non-infectious inflammatory conditions
	Recent surgery, myocardial ischemia within 3 months

CT, computerized tomography; MRI, Magnetic resonance imaging.

### Blood draw and interinstitutional transportation

Venous blood samples were collected by venipuncture immediately following informed consent and enrollment. The blood sample was collected within 0–48 h of symptom onset. Five milliliters of blood were placed in an EDTA-vacutainer and centrifuged at 1,500 × g for 15 mins. The supernatant plasma was aliquoted in sterile 1 ml cryotubes and stored at −80°C until analysis. Frozen plasma samples were transported in a sealed box with dry ice from OMC to Marshall University, taking standard precautions in the transportation of biological material, for subsequent processing and analysis. All precautionary and safety measures in handling and transporting potentially biohazardous specimens were employed.

### Clinical data collection

#### ICH volume

The volume of ICH on computed tomography scan was calculated using the validated formula ABC/2 and was analyzed as a continuous variable and stratified as previously described as <30 ml. 30–60 ml, and >60 ml as a predictor of outcome and severity (Broderick et al., 1993; Hemphill et al., 2001, 2015; Qureshi et al., 2001).

#### NIHSS

The neurological deficits can be quantitatively measured by the national institutes of health stroke scale (NIHSS) worldwide. It is a questionnaire with a total of 11 items, a picture, and words to read, structured to determine the level of stroke in an individual.

#### ICH severity and outcomes

Our outcome measure was Modified Rankin Scale (MRS) at 90 days. At 90 days, functional outcome evaluation was performed by an MRS-certified clinical research coordinator at OMC. In addition to hematoma volume as described above, the previously validated Hemphill (ICH) score was used as a measure of ICH severity (range 0–6) (Hemphill et al., 2001, 2009).

#### Data collection

All enrolled subjects have had data collected based on the stroke common data (CDE) elements recommended by the National Institute of Neurological Diseases and Stroke (NINDS). Data collected were stored using a Redcap^®^ database and maintain HIPAA compliance. An identification number was assigned to each enrolled participant at the time of informed consent to ensure privacy and confidentiality for the duration of the study. All data were de-identified for data analysis.

### lEV and sEV isolation

lEV/sEV were isolated by differential centrifugation method (Wang et al., 2016a,b). Collected plasma samples were centrifuged at 300×g for 15 min, followed by centrifugation at 2,000×g for 30 min to remove cells and cell debris. Then the cell-free plasma was centrifuged at 20,000×g for 70 min to isolate pellets of lEVs. The supernatant is ultracentrifuged at 170,000 g for 1.5 h to isolate sEVs pellets by using a Sorvall MX 120 Plus micro-ultracentrifuge with a S110-AT fixed angle rotor. The pelleted lEV/sEV were resuspended with 20 nm filtered (Whatman, PA) phosphate-buffered saline (PBS).

### Measurements of circulating lEV/sEV levels and phenotypes

The levels and the phenotypes of the circulating lEV/sEV were measured by using the cell-specific antibodies combining Nanoparticle Tracking System (NTA) per our previous publications (Wang et al., 2016a,b). Specifically, lEV/sEV were diluted and incubated with primary antibodies—CD66b (1 mg/ml, Invitrogen), P2RY12 (1 ug/ul, Abnova), and CD80 (1.891 mg/ml, Abcam), for 2 h followed by secondary antibody—Qdot 655 for 90 min. NTA (NanoSight NS300, Malvern Panalytical, Amesbury, UK) was used to measure the concentration and particle size of lEV and sEV. They were detected under the fluorescence mode after labeling with cell-specific antibodies. The concentration of lEV/sEV was defined as the number of particles per 1 ml plasma. The percentage of CD66b, P2RY12 and CD80 positive lEV/sEV was determined: (the concentration of CD66b, P2RY12 and CD80 positive lEV/sEV)/(concentration of total circulating lEV/sEV) × 100.

### Statistical analysis

The paired comparison was analyzed using Student's t-test. The comparison of multiple groups was detected by one-way or two-way ANOVA with a Tukey or other appropriate *post-hoc* test. Associations between different variables were investigated with Spearman rank correlation. Analysis of multiple variables and continuous responses were considered using ANOVA and multiple linear regression with *F*-statistics and *p*-values presented to show statistical significance relative to hypothesis tests that the model of selected variables does not have a relationship with the dependent variable. Stepwise regression, consistent with, was used to develop parsimonious multiple linear regression models wherein variables were removed sequentially in a backward manner based on minimum Bayesian Information Criterion (BIC) (Kutner, 2005). Statistical calculations were performed using JMP 14 (SAS, Cary, NC) as well as GraphPad Prism. *p* < 0.05 was considered statistically significant, and raw *p*-values from the underlying statistical tests were presented since many *p*-values were considerably smaller than 5%. Due to the clinical nature of the study, significance at the 10% level is indicated in cases where a statistical hypothesis was not rejected but the *p*-values were <10%.

## Results

### The concentration of circulating lEV and sEV is increased in the patients with higher NIHSS and larger ICH volume

**Figure 1:**
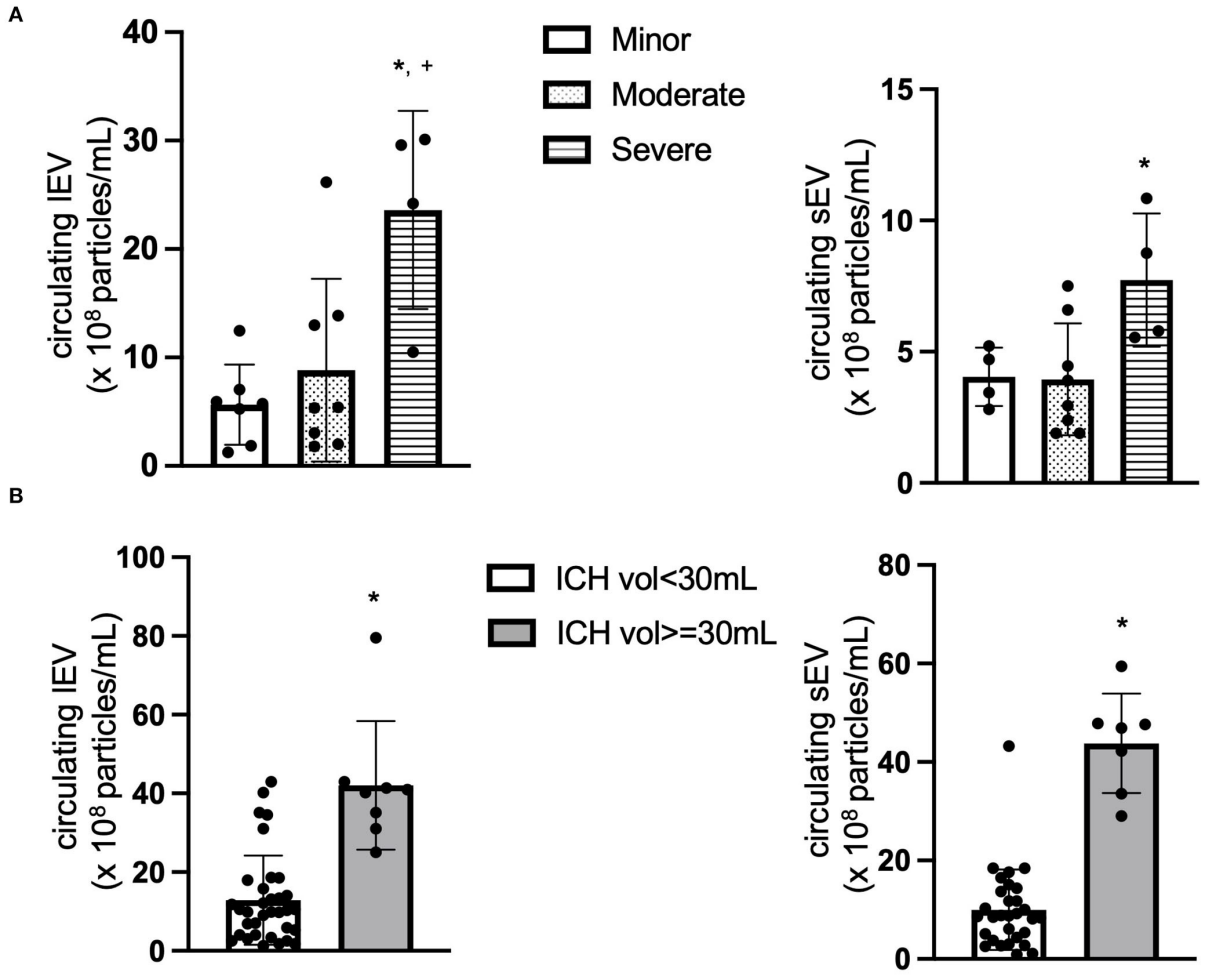
The concentration of circulating lEV and sEV. **(A)** The concentration (×10^8^ particles/ml plasma) of circulating lEV and sEV according to NIHSS. *p < 0.05 vs. minor, ^+^*p* < 0.05 vs. Moderate. *n* = 36. **(B)** The concentration (×10^8^ particles/ml plasma) of circulating lEV and sEV according to ICH volume. *p < 0.05 vs. ICH volume < 30 ml. *n* = 39.

First of all, the total levels of circulating lEV and sEV from the ICH patients were measured and compared based on the disease severity according to NIHSS. The patients were divided into three different groups: minor (1–4), moderate (5–20) and severe (21–42) based on their NIHSS. As shown in Figure 1A, the concentrations of circulating lEV (*F* = 8.42, *p* < 0.05, Severe vs. Minor or Moderate) and sEV (*F* = 5.028, *p* < 0.05, Severe vs. Moderate) were increased in the group of patients with higher NIHSS.

We also compared the level of circulating lEV and sEV based on the disease severity according to ICH volume, ICH volume < 30 ml and ICH volume ≥ 30 ml. As shown in Figure 1B, the concentrations of circulating lEV (*t* = 6.008, *p* < 0.05, vs. ICH Volume < 30 ml) and sEV (t = 9.457, *p* < 0.05, vs. ICH volume < 30 ml) were increased in the group of patients with larger ICH volume.

### The concentration of CD66b+ lEV is increased whereas the level of CD66b+ sEV is decreased with patients having higher NIHSS

**Figure 2:**
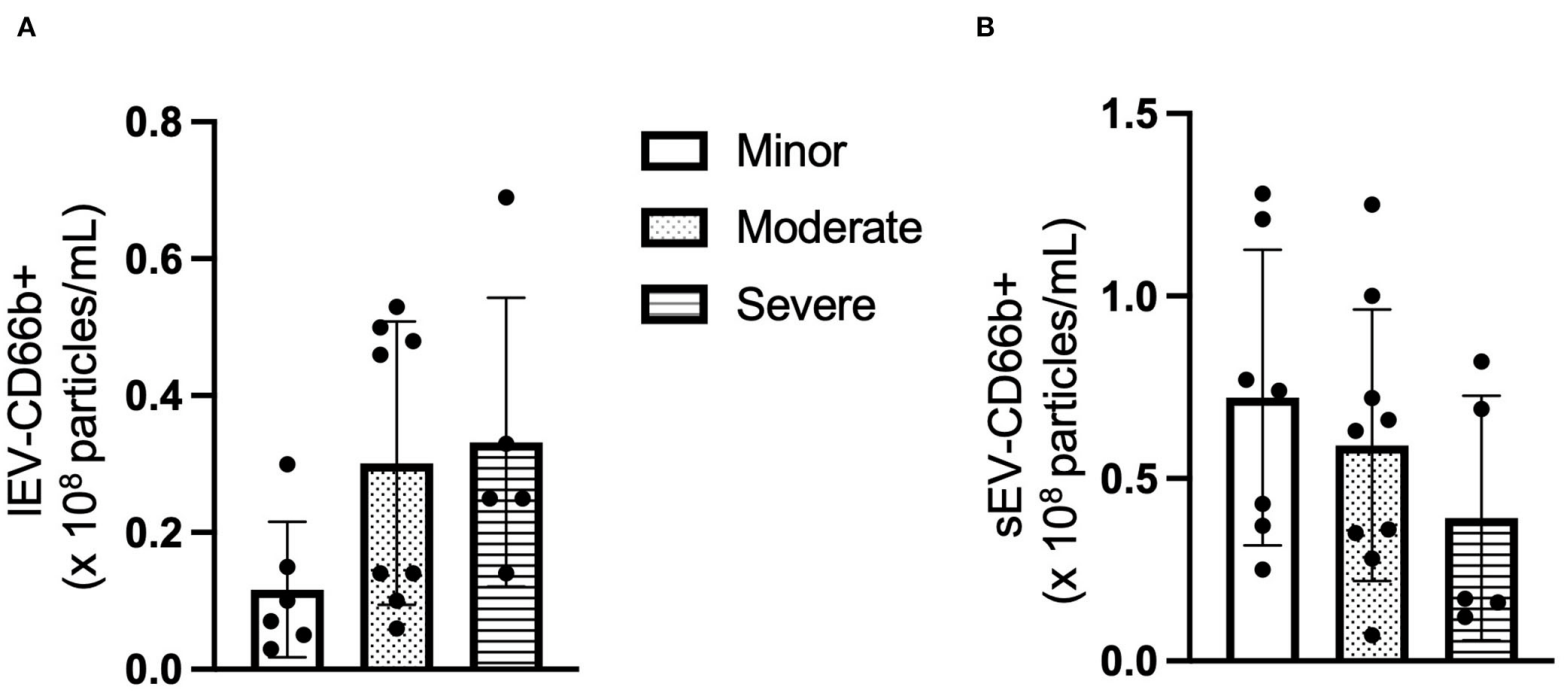
The concentration of CD66+ EV according to NIHSS categories. **(A)** The concentration of CD66+ lEV according to NIHSS categories. **(B)** The concentration of CD66+ sEV according to NIHSS categories. *n* = 36. The unit for the concentration is ×10^8^ particles/ml plasma.

Next, the cell origin of the circulating lEV and sEV was determined by using the cell-specific antibodies. The data from NTA showed that the concentration of CD66+ lEV was increased in the patients with higher NIHSS (*F* = 2.447, *p* > 0.05, Figure 2A), whereas the concentration of CD66b+ sEVs was decreased in the patients with higher NIHSS (*F* = 1.124, *p* > 0.05, Figure 2B).

### The ratio of P2RY12/CD80+ LEVs is increased whereas it decreased in P2RY12/CD80+ sEV with patients having higher NIHSS

**Figure 3:**
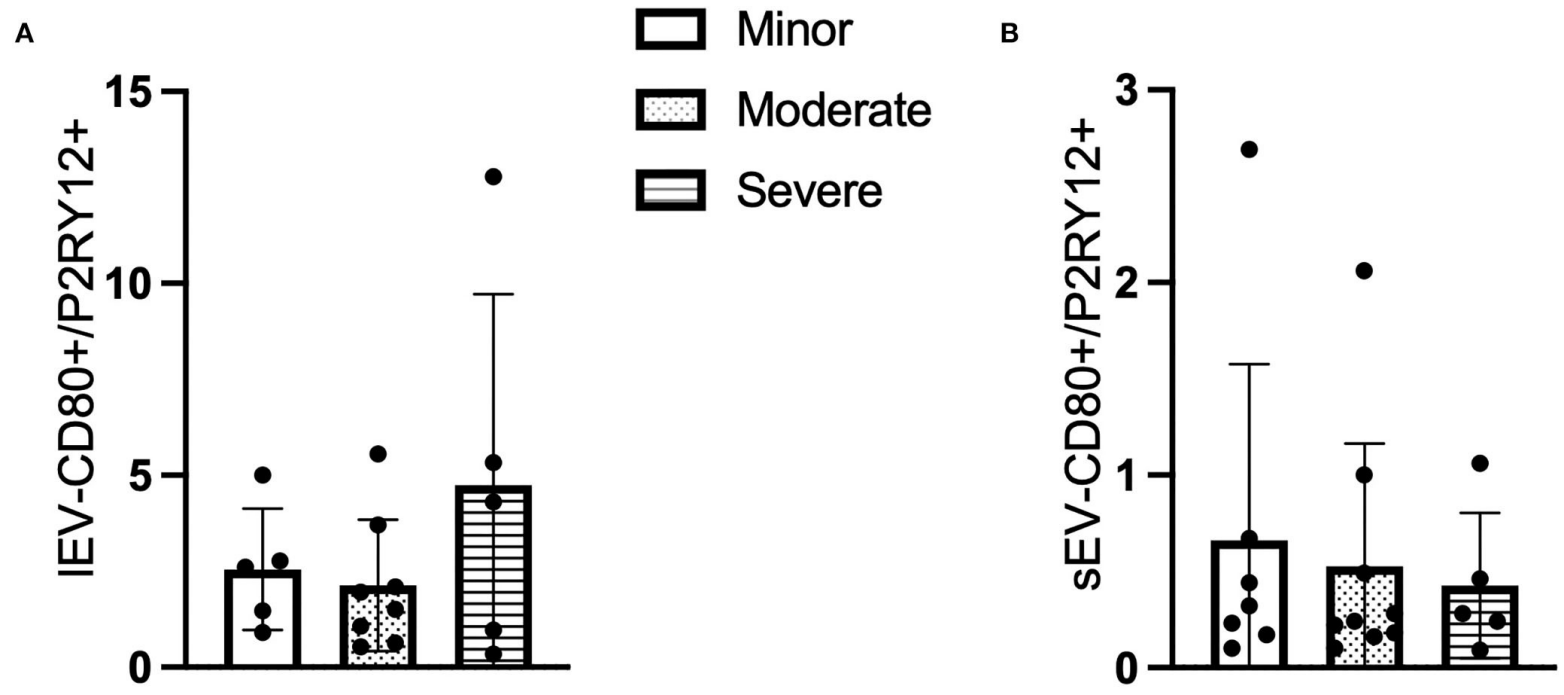
The ratio of CD80+ and P2RY12+ EV according to NIHSS categories. **(A)** The ratio of CD80+/P2RY12+ lEV according to NIHSS categories. **(B)** The ratio of CD80+/P2RY12+ sEV according to NIHSS categories. n = 36.

Similar to the CD66b+ lEV/sEV data, the ratio of circulating *P2RY12/CD80*+ lEV was increased in the patients with higher NIHSS (*F* = 1.294, *p* > 0.05, Figure 3A), whereas the concentration of *P2RY12/CD80*+ sEV is decreased in the patients with higher NIHSS (*F* = 0.169, *p* > 0.05, Figure 3B).

### Correlations of the concentration lEV and sEV with the clinical parameters

Of the 39 patients [22 (M), 17 (F); 58 (15) years] who were treated for ICH had a mean hospital length of stay of 15 (12) days and mean intensive care unit (ICU) length of stays of 10 (10) days. The ICH volume for the patients had a mean of 21 (27) at admission and 23 (28) 24 h after admission.

**Figure 4:**
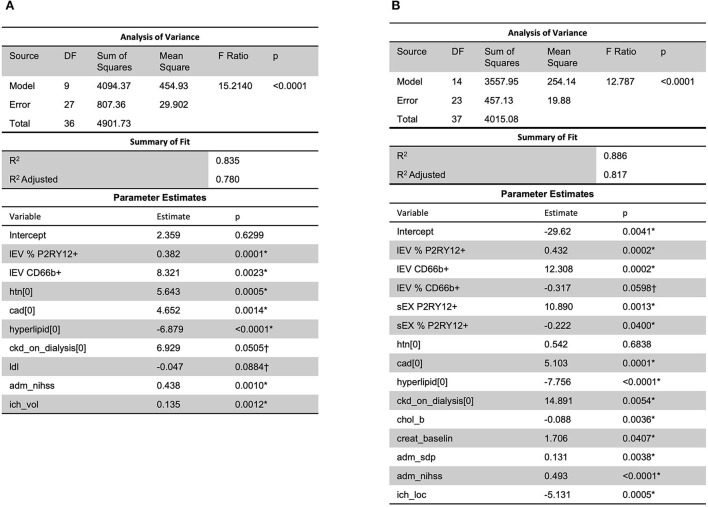
A predictive relationship between both **(A)** hospital length of stay (*R*^2^ = 0.83) and **(B)** ICU length of stay (*R*^2^ = 0.88) was found with lEV and sEV and patient data (including LDL, ICH volume, etc.). One outlier was removed in **(A)** to reduce non-constant error variance. *Significance at the 5% level, ^†^Significance at the 10% level. LDL, low-density lipoprotein.

Analysis using stepwise regression for hospital length of stay (LOS) as a dependent variable and all other variables, excluding LOS variables, as independent variables produced the model in Figure 4A. This model was statistically significant (*p* < 0.0001) and explained a majority of the variation in the data (*R*^2^ = 0.83); thus indicating that there was a statistically significant predictive relationship between the clinical outcome of hospital LOS and these variables. Further analysis using stepwise regression with ICU LOS as the dependent variable and the other variables in the data, excluding LOS variables, as independent variables produced the model in Figure 4B. This model was statistically significant (*p* < 0.0001) and explained most of the variation in the data (*R*^2^ = 0.88).

**Figure 5:**
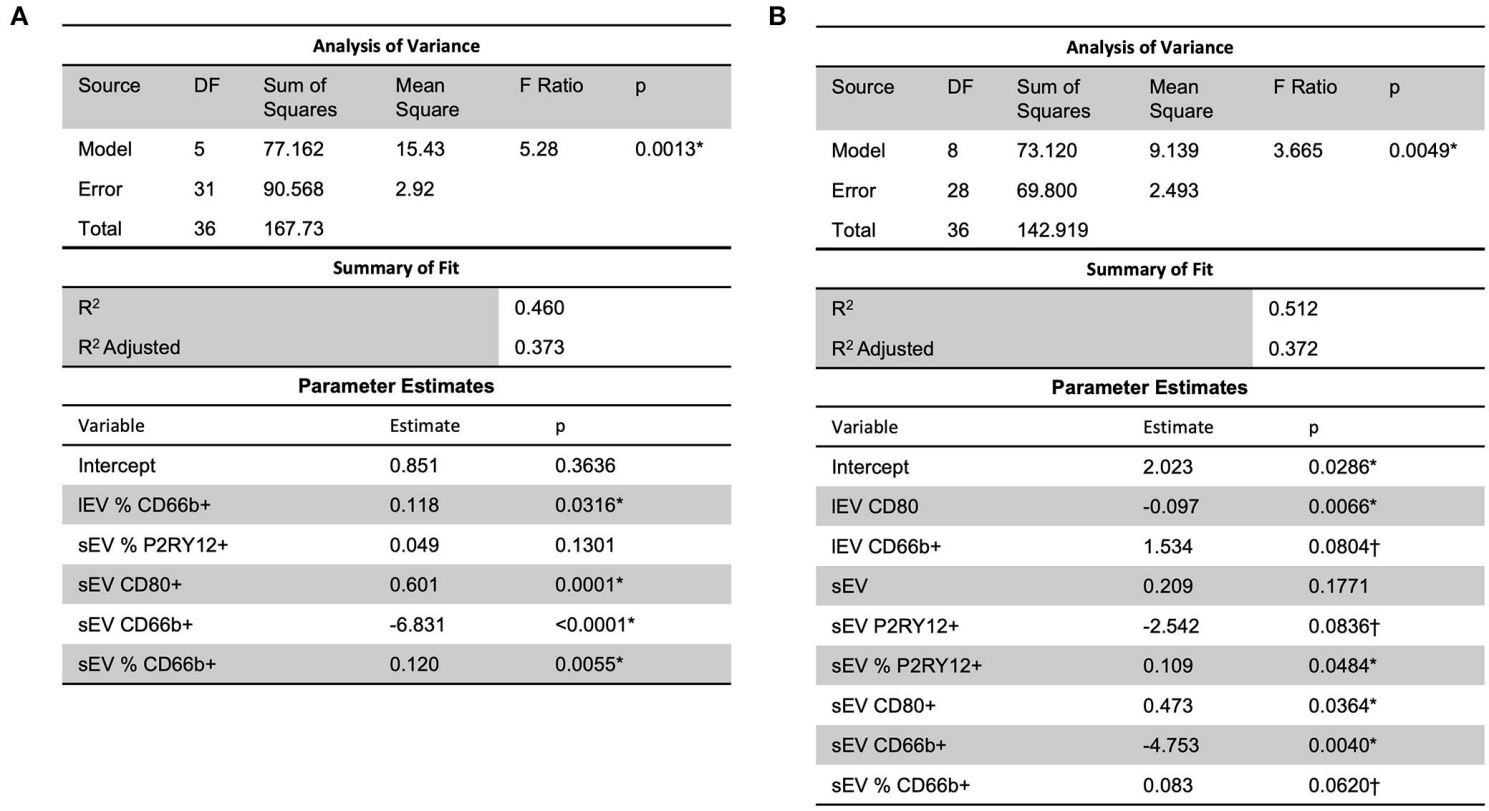
A predictive multiple linear regression relationship was seen between lEV and sEV concentrations and MRSV3 (*R*^2^ = 0.46) and MRSV5 (*R*^2^ = 0.51), however, some issues existed with non-constant error variance in these models. One outlier was removed in **(A,B)** to reduce non-constant error variance. *Significance at the 5% level, ^†^Significance at the 10% level.

Analysis using stepwise regression between MRSV3 (Modified Rankin Scale at 90 days) as a dependent variable and independent variables being the lEV and sEV expression produced the model in Figure 5A which was statistically significant (*p* < 0.0013, *R*^2^ = 0.46). A similar analysis using stepwise regression with MRSV5 (Modified Rankin Scale at 180 days) as a dependent variable and independent variables being the lEV and sEV expression produced a statistically significant relationship (*p* < 0.0049, *R*^2^ = 0.51) as seen in Figure 5B. However, some issues existed with non-constant error variance in the models in Figure 5, this is likely due to the small clinical sample size and a larger study is needed to further study this relationship.

### Slight predictive relationship between lEV and sEV expression and hospital/ICU length of stay

**Figure 6:**
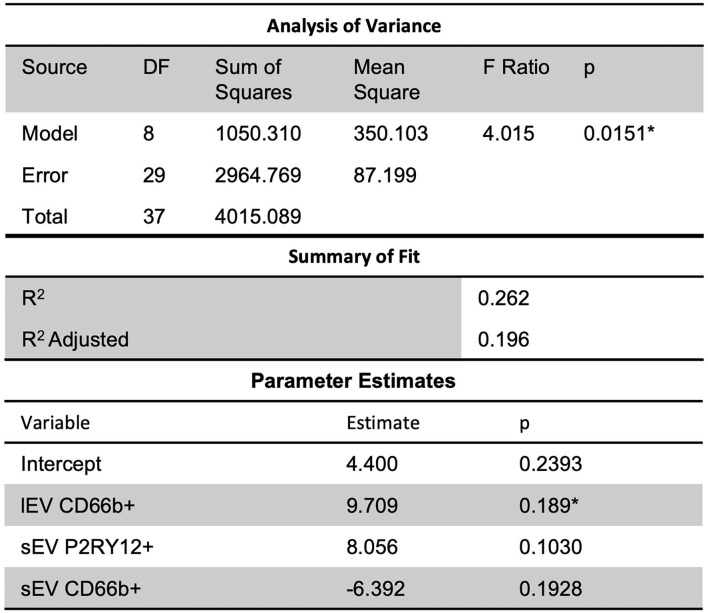
Further, slight predictive relationships were seen between lEV and sEV concentrations and ICU length of stay (*R*^2^ = 0.26). However, the model had insignificant terms and non-constant error variance issues due to the small sample size and outliers. *Significance at the 5% level.

Further, a slight predictive multiple linear regression relationship (*R*^2^ = 0.26) was seen, using a stepwise regression approach, between ICU LOS and independent variables of lEV and sEV concentrations, CD66b+ lEV, P2RY12+ sEV, and CD66b+ sEV (Figure 6); while the correlation was slight, the model was statistically significant (*p* = 0.0151). However, this model had insignificant terms and non-constant error variance issues due to the small sample size and outliers. This issue is likely due to the small sample size in this study and thus performing a similar, but a larger study is needed to further study this relationship.

### Clinical response is predictable given the combination of lEV and sEV concentrations and other health conditions

**Figure 7:**
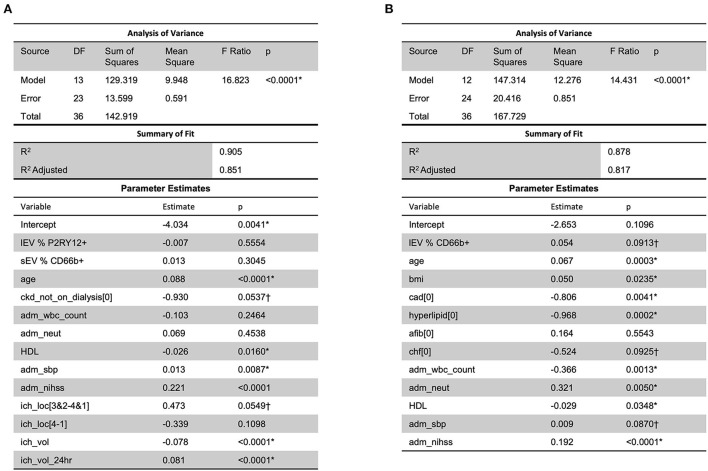
When considering all collected data with MRS V3 and MRS V5, a predictive relationship was found through stepwise regression with MRS V3 (*R*^2^ = 0.90) and MRS V5 (*R*^2^ = 0.87). One outlier was removed in **(A,B)** to reduce non-constant error variance. *Significance at the 5% level, ^†^Significance at the 10% level.

When considering MRSV3 as a dependent variable and all collected data as independent variables using a stepwise approach, the model in Figure 7A was produced. This model has a statistically significant multiple linear regression predictive relationship between these variables and MRSV3 (*p* < 0.0001, *R*^2^ = 0.90). When a similar stepwise regression approach was used with MRSV5 as a dependent variable and other all collective data variables as independent variables using a stepwise approach, the model in Figure 7B was produced. This model has a statistically significant multiple linear regression predictive relationship between the variables in the parameter estimate table and MRSV5 (*p* < 0.0001, *R*^2^ = 0.87).

## Discussion

Tissue or cell-specific lEV and sEV have been used as biomarkers for various diseases (Wang et al., 2016a; Nik Ibrahim et al., 2022). Our group has established an NTA method to detect the level of circulating endothelial lEV/sEV which could be a biomarker of outcome for ischemic stroke. In the previous study, we found that the levels of circulating endothelial cells and endothelial progenitor cells-released sEV (exosomes) on day 3 and day 5 were increased when compared to day 1 after ischemic stroke, which could be potential biomarkers for the disease prognosis in ischemic stroke (Wang et al., 2016a). However, there is no specific biomarker of neuroinflammation to predict the outcome of ICH or to guide the treatment of inflammation during the ICH. In the current study, we determined the levels and the phenotypes of circulating lEV/sEV in ICH patients and the correlation with the disease outcome and severity. This study is the first study to determine the circulating levels and cell origin of lEV/sEV in ICH patients and analyze the correlation of circulating lEV/sEV or cell-specific lEV/sEV with the outcome of ICH.

It should be noted that the macrophage is a key factor of neuroinflammation that is crucial to brain injury as well as recovery after ICH. The inflammatory processes involve the activation of local macrophages (microglia) and leucocyte-derived macrophage infiltrated from the circulation (Xue and Yong, 2020). Previous studies demonstrate that macrophages can play double roles after stroke through a pro-inflammatory or regulatory phenotype. The pro-inflammatory macrophages produce injury-enhancing factors, while the regulatory macrophages produce potential reparative and anti-inflammatory factors (Bai et al., 2020). As a prominent driver of secondary injury, neuroinflammation is a key factor for brain injury and recovery of ICH. Knowing the status of neuroinflammation is particularly useful for predicting the prognosis and guiding the treatment of ICH. In this regard, the phenotype of macrophages could be a useful biomarker of the disease prognosis and treatment efficacy. However, because of the BBB, large molecules are not easy to pass through and be detected in the circulation. EVs are emerging as novel communicators between cells and organs. They can be released from all cells and carry the molecular contents of the parent cells (van Niel et al., 2018). More importantly, EVs are nanosized and could cross the BBB (Jin T. et al., 2021). These properties make EVs the perfect candidates for biomarkers of neuroinflammation after ICH.

First of all, we measured the level of circulating total lEV and sEV in the plasma of ICH patients. Based on the ICH severity (ICH volume and NIHSS), the patient population was divided into different groups. The data showed that the concentrations of circulating lEV/sEV were increased in the subpopulation with higher NIHSS and larger ICH volume. This data suggests that the circulating level of lEV/sEV could be a biomarker for the disease progress of ICH. As we know that different risk factors, such as stress, cell activation or apoptosis, could promote the release of lEV/sEV. Therefore, the increased level of circulating lEV/sEV levels could be from the activated circulating cells after ICH, including monocytes, neutrophils, platelet, etc. To determine which cell type is involved, the cell-specific antibodies combined with the NTA system were applied. Since inflammatory cells play important roles in ICH, we measured the phenotypes and the level of lEV/sEV derived from different leukocytes (neutrophils, microglia, and macrophages) by using the cell-specific antibodies (CD66b, P2RY12 and CD80) in the present study. Neutrophils overexpressed CD66b after activation during acute infection (Fortunati et al., 2009). CD66b is a marker of granulocyte activation involved in adhesion to endothelial cells, degranulation, and increased reactive oxygen species (ROS) production (Opasawatchai et al., 2019). P2RY12 is found mainly but not exclusively on the surface of blood platelets, and is an important regulator in blood clotting (Dorsam and Kunapuli, 2004). In the central nervous system, this receptor has been found expressed exclusively on microglia, where it is necessary for physiological and pathological microglial actions, such as monitoring neuronal functions and microglial neuroprotection (Cserép et al., 2020). CD80 is present specifically on the surface of various immune cells including activated B-cells, and macrophages, T-cells. CD80 has a crucial role in modulating T-cell immune (Peach et al., 1995). We found that the CD66b+ lEV and the P2RY12/CD80+ lEV were increased in the subpopulation with higher NIHSS, while their sEV-levels were decreased in the subpopulation with higher NIHSS. This is very interesting data since the lEV are released mostly from the membrane budding, while the sEV are released through exocytosis. Accordingly, the release of lEV could be earlier than the sEV. This could be the reason that we observed the lEV level was increased since the blood samples were collected in the acute phase (within 3 days of onset). However, larger data and more time points are needed to confirm the changes. In addition, there are no differences between the group with ICH volume > 30 ml and ICH volume < 30 ml in the CD66b+/P2RY12+/CD80+- lEV and sEV levels (data not shown). Further study is needed to define the cell origin of lEV and sEV by using multiple cell-specific markers.

To determine the role of circulating lEV/sEV and inflammation-related lEV/sEV in the progress of ICH, correlation and regression analyses were performed between the levels of total circulating lEV/sEV and cell-specific lEV/sEV with the clinical data, such as low-density lipoprotein (LDL), systolic blood pressure, hospital length of stay or ICU length of stay, etc. A predictive relationship between both hospital length of stay and ICU length of stay was found with lEV and sEV and patient data (including LDL, ICH volume, etc.). Further predictive multiple linear regression relationship was seen between lEV and sEV concentrations and MRSV3 (*R*^2^ = 0.46) and MRSV5 (*R*^2^ = 0.51), however, some issues existed with non-constant error variance in these models. Further, slight multiple linear regression relationships were seen between lEV and sEV concentrations and both hospital length of stay and ICU length of stay. When considering all collected data with MRS V3 and MRS V5, a predictive relationship was found through stepwise regression with MRS V3 (*R*^2^ = 0.90) and MRS V5 (*R*^2^ = 0.87). However, models had insignificant terms and non-constant error variance issues due to small sample size and outliers.

## Conclusions and limitations

This study found predictive relationships between patient outcomes and lEV and sEV. When combined with generally collected patient data (ICH volume, etc.), measurements of lEV and sEV are strongly predictive of overall patient outcome. However, this study is limited by its small sample size and imbalanced data. Further, a larger study is needed to investigate these effects. In addition, more or multiple cell-specific markers are needed to define the cell origin of lEV and sEV. For examples, the lEV and sEV released from the neutrophils, macrophages, endothelial cells, endothelial progenitor cells, platelet, etc.

## Perspectives

The completion of this study has identified the phenotype of circulating lEV/sEV and defined the potential biomarker role of circulating lEV/sEV in ICH. The results of this project could subsequentially lead to the discovery of therapeutic strategies for treating stroke patients and improving the life quality of stroke patients and subsequently reducing the mortality of stroke and the economic burden from strokes.

## Data availability statement

The original contributions presented in the study are included in the article/supplementary material, further inquiries can be directed to the corresponding author.

## Ethics statement

The studies involving human participants were reviewed and approved by Ochsner Medical Center's Institutional Review Board (IRB, #2015.137.A). The patients/participants provided their written informed consent to participate in this study.

## Author contributions

JB and II designed and supervised the research, interpreted the data, and drafted the manuscript. II and DN provided the patients' samples and the clinical data. HS performed the research, collected and analyzed data, and made the figures. TB performed the statistical analysis. II, TB, and DN revised and approved the manuscript. JB drafted the manuscript. All authors contributed to the article and approved the submitted version.

## Funding

This work was supported by the American Heart Association (16SDG26420078), the National Institute of General Medical Sciences (U54GM104942), and the National Institute of Neurological Disorders and Stroke (1R01NS102720). Ochsner Clinic Foundation.

## Conflict of interest

The authors declare that the research was conducted in the absence of any commercial or financial relationships that could be construed as a potential conflict of interest.

## Publisher's note

All claims expressed in this article are solely those of the authors and do not necessarily represent those of their affiliated organizations, or those of the publisher, the editors and the reviewers. Any product that may be evaluated in this article, or claim that may be made by its manufacturer, is not guaranteed or endorsed by the publisher.
